# Multiscale Cross-Approximate Entropy Analysis as a Measure of Complexity among the Aged and Diabetic

**DOI:** 10.1155/2013/324325

**Published:** 2013-06-20

**Authors:** Hsien-Tsai Wu, Cyuan-Cin Liu, Men-Tzung Lo, Po-Chun Hsu, An-Bang Liu, Kai-Yu Chang, Chieh-Ju Tang

**Affiliations:** ^1^Department of Electrical Engineering, National Dong Hwa University, No. 1, Section 2, Da Hsueh Road, Shoufeng, Hualien 97401, Taiwan; ^2^Research Center for Adaptive Data Analysis & Center for Dynamical Biomarkers and Translational Medicine, National Central University, Chungli 32001, Taiwan; ^3^Department of Neurology, Buddhist Tzu Chi General Hospital and Buddhist Tzu Chi University, Hualien 97002, Taiwan; ^4^Department of Internal Medicine, Hualien Hospital, Health Executive Yuan, Hualien 97061, Taiwan

## Abstract

Complex fluctuations within physiological signals can be used to evaluate the health of the human body. This study recruited four groups of subjects: young healthy subjects (Group 1, *n* = 32), healthy upper middle-aged subjects (Group 2, *n* = 36), subjects with well-controlled type 2 diabetes (Group 3, *n* = 31), and subjects with poorly controlled type 2 diabetes (Group 4, *n* = 24). Data acquisition for each participant lasted 30 minutes. We obtained data related to consecutive time series with R-R interval (RRI) and pulse transit time (PTT). Using multiscale cross-approximate entropy (MCE), we quantified the complexity between the two series and thereby differentiated the influence of age and diabetes on the complexity of physiological signals. This study used MCE in the quantification of complexity between RRI and PTT time series. We observed changes in the influences of age and disease on the coupling effects between the heart and blood vessels in the cardiovascular system, which reduced the complexity between RRI and PTT series.

## 1. Introduction

Multiple temporal and spatial scales produce complex fluctuations within the output signals of physiological systems [1]. In recent studies on translational medicine [1–5], researchers have found that implicit information within the complex fluctuations of physiological signals can be used to evaluate health conditions.

 Many recent studies [2, 3] have employed nonlinear dynamical analysis to quantify the complexity of physiological signals in the cardiovascular system. Costa et al. [2] were the first to propose multiscale entropy (MSE) as an approach to analyze the R-R interval (RRI) series of healthy individuals and discovered that the RRI series of young individuals were more complex than that of elderly people. Wu et al. [3] adopted the same method in an examination of pulse wave velocity (PWV) and found that the complexity of these series decreased with aging and/or the progression of diabetes. In addition to time and space, “coupling behavior” in the physiological system also affects the complexity of individual physiological signals, such as RRI or PWV [6]. Drinnan et al. [7] indicated that pulse transit time (PTT) is influenced by RRI and other cardiovascular variables and used cross-correlation functions to quantify the phase relationship between the two time series signals in the cardiovascular system. They established that there was a strong correlation between PTT and RRI variations in healthy subjects. However, Pincus [8] claimed that cross-approximate entropy (Co_ApEn) is more effective than cross-correlation functions in the evaluation of complexity between the two series.

Despite the fact that Co_ApEn has been widely applied to evaluate the complexity between two time series [9–12], single-scale entropy values are not necessarily able to identify the dynamic complexity of physiological signals. Therefore, this study was an attempt to use a multiscale Co_ApEn (MCE) [13] to quantify the complexity between the synchronous time series of cardiac functions and the degree of atherosclerosis. We assumed that complexity would exist in RRI and PTT series of the cardiovascular system due to the mutual interaction between the heart and blood vessels. Moreover, we assumed that complexity reduces with aging and the influence of disease. We used MCE to develop an index for the quantification of complexity between the two time series capable of distinguishing between healthy individuals and those with diabetes.

## 2. Methods

### 2.1. Study Design

This study evaluated the influences of age and diabetes on RRI and PTT. Considering that RRI and PTT are nonlinear, cardiovascular variables, we tested the applicability of MCE in the study subjects and investigated whether this dynamic parameter could provide further information related to the clinical control of diabetes.

### 2.2. Subject Populations and Experiment Procedure

Between July 2009 and March 2012, four groups of subjects were recruited for this study: young healthy subjects (Group 1, age range: 18–40, *n* = 32), healthy upper middle-aged subjects (Group 2, age range: 41–80, *n* = 36), subjects with well-controlled type 2 diabetes (Group 3, age range: 41–80, *n* = 31, 6.5% ≦ glycosylated hemoglobin (HbA1c) < 8%), and subjects with poorly controlled type 2 diabetes (Group 4, age range: 41–80, *n* = 24, HbA1c ≧ 8%) [3]. The other 22 subjects were excluded due to incomplete or unstable waveform data acquisition. All diabetic subjects were recruited from the Hualien Hospital Diabetic Outpatient Clinic; healthy controls were recruited from a health examination program at the same hospital. None of the healthy subjects had personal or family history of cardiovascular disease. Type 2 diabetes was diagnosed as either fasting sugar higher than 126 mg/dL or HbA1c ≧ 6.5%. All diabetic subjects had been receiving regular treatment and follow-up care in the clinic for more than two years. Regarding the use of medications, there was no significant difference in the type (i.e., antihypertensive, lipid-lowering, and hypoglycemic medications), dosage, and frequency among the well-controlled and poorly controlled diabetic subjects. This study was approved by the Institutional Review Board (IRB) of Hualien Hospital and National Dong Hwa University. All subjects refrained from caffeinated beverages and theophylline-containing medications for 8 hours prior to each hospital visit. Each subject gave informed consent, completed questionnaires on demographic data and medical history, and underwent blood sampling prior to data acquisition. Blood pressure was obtained once from the left arm of supine subjects using an automated oscillometric device (BP3AG1, Microlife, Taiwan) with a cuff of appropriate size, followed by the acquisition of waveform data from the second toe using a six-channel ECG-PWV [14, 15] as previously described.

### 2.3. Data Collection and Calculation of RRI and PTT Series

**Figure 1:**
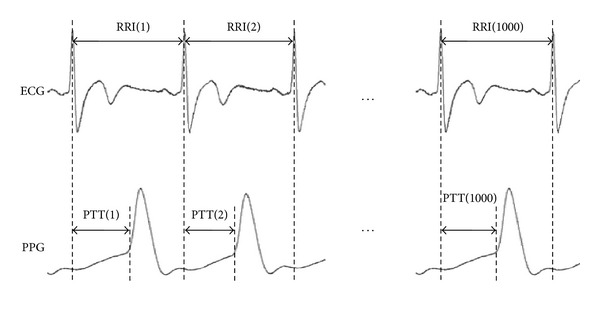
1000 consecutive data points from ECG signals and PPG signals: PTT(*j*) refers to the time interval between the R-wave peak of the *j*th cardiac cycle to the footpoint of the toe pulse from the left foot.

All subjects were permitted to rest in a supine position in a quiet, temperature-controlled room at 25 ± 1°C for 5 minutes prior to subsequent 30-minute measurements. Again, a good reproducibility of six-channel ECG-PWV system [14, 15] was used for waveform measurement from the second toe. Infrared sensors were simultaneously applied to points of reference for the acquisition of data. Electrocardiogram (ECG) measurements were obtained using the conventional method. After being processed through an analog-to-digital converter (USB-6009 DAQ, National Instruments, Austin, TX USA) at a sampling frequency of 500 Hz, the digitized signals were stored on a computer. Because of its conspicuousness, the R wave in Lead II was selected as a reference point: the time interval between the R-wave peak of the *j*th cardiac cycle to the footpoint of the toe pulse from the left foot was defined as PTT(*j*); the time difference between the two continues peak of ECG R wave was defined as RRI(*i*), as shown as Figure 1.

Using ECG and photoplethysmography (PPG), we obtained the RRI series {RRI(*i*)} = {RRI(1), RRI(2),…, RRI(1000)} and PTT series {PTT(*j*)} = {PTT(1), PTT(2),…, PTT(1000)} from each subject. All series were retrieved from 1000 consecutive, stable ECG tracings and PPG toe pulse signals synchronous with the cardiac cycle [14].

Due to a trend within physiological signals [6, 16], nonzero means may be included; therefore, we used empirical mode decomposition (EMD) [17] to deconstruct the {RRI(*i*)} and {PTT(*j*)} series, thereby eliminating the trend from the original series. We then normalized the {RRI(*i*)} and {PTT(*j*)} series, as shown in (1). In these equations, SD_*x*_ and SD_*y*_ represent the standard deviations of series {RRI(*i*)} and {PTT(*j*)}, respectively. Complexity analysis was performed on the normalized results, {RRI′(*i*)} and {PTT′(*j*)}. Consider

(1) $$\begin{array}{c} \left\{\text{RR}{\text{I}}^{\prime }\left(i\right)\right\}=\frac{\left\{\text{RRI}\left(i\right)\right\}}{\text{S}{\text{D}}_{x}},\\ \left\{{\text{PTT}}^{\prime }\left(j\right)\right\}=\frac{\left\{\text{PTT}\left(j\right)\right\}}{\text{S}{\text{D}}_{y}}. \end{array}$$

### 2.4. Multiscale Cross-Approximate Entropy (MCE) Using Normalized RRI and PTT Series Together

Previous studies [1–3, 18] have employed MSE to overcome comparison difficulties at a scale factor of 1, when physiological complexity is reduced due to age or disease. However, other research [7] has indicated a strong relationship between variations in PTT series and RRI series; therefore, we used MCE to investigate the interactions between PTT and RRI.

#### 2.4.1. Coarse-Grained Process and Cross-Approximate Entropy (Co_ApEn)

MSE involves the use of a scale factor *τ* (*τ* = 1, 2, 3,…, *n*), which is selected according to a 1D series of consecutive cycles. This factor enables the application of a coarse-graining process capable of deriving a new series prior to the calculation of entropy in each new individual series [1–3, 18]. Using this approach, we performed coarse-graining on the normalized 1D consecutive cycles of the {RRI′(*i*)}  and  {PTT′(*j*)} series based on scale factor *τ*, thereby obtaining the series {RRI^′(*τ*)^} and {PTT^′(*τ*)^} as shown in (2). We then calculated entropy as follows:

(2) $$\begin{array}{c} \text{RR}{\text{I}}^{\prime }{\left(u\right)}^{\left(\tau \right)}=\frac{1}{\tau }\sum_{i=\left(u-1\right)\tau +1}^{u\tau }\text{RR}{\text{I}}^{\prime }\left(i\right),\;1\leq u\leq \frac{1000}{\tau },\\ \text{PT}{\text{T}}^{\prime }{\left(u\right)}^{\left(\tau \right)}=\frac{1}{\tau }\sum_{j=\left(u-1\right)\tau +1}^{u\tau }\text{PT}{\text{T}}^{\prime }\left(j\right),\;1\leq u\leq \frac{1000}{\tau }. \end{array}$$

Previous studies [19, 20] have used Co_ApEn, an improved analysis method of approximate entropy, to analyze two synchronous physiological time series, define their relationship, and calculate the complexity within that relationship [8, 21]. This method utilizes the dynamic changes between the two series to evaluate the physiological system. Similarities between changes in the two series can be used to observe the regulatory mechanisms in the physiological system. However, many studies [8, 19–21] presented their results at a scale factor of 1. To obtain a deeper understanding of the complexity of the physiological system, we utilized coarse-grained {RRI^′(*τ*)^} and {PTT^′(*τ*)^} series to calculate the Co_ApEn at each scale, using (7). We refer to this approach as multiscale cross-approximate entropy (MCE). The details of the algorithm are as follows [22].

(1) For given *m*, for two sets of *m*-vectors,
  (3) x(i)≡[RRI′(τ)(i)RRI′(τ)(i+1)⋯RRI′(τ)(i+m−1)],i=1, N−m+1,y(j)≡[PTT′(τ)(j)PTT′(τ)(j+1)⋯PTT′(τ)(j+m−1)],j=1, N−m+1.
(2) Define the distance between the vectors **x**(*i*), **y**(*j*) as the maximum absolute difference between their corresponding elements, as follows:
  (4) d[x(i),y(j)] =max⁡k=1m[|RRI′(τ)(i+k−1)−PTT′(τ)(j+k−1)|].
(3) With the given **x**(*i*), find the value of *d*[**x**(*i*), **y**(*j*)] (where *j* = 1 to *N* − *m* + 1) that is smaller than or equal to *r* and the ratio of this number to the total number of *m*-vectors (*N* − *m* + 1). That is,
   let *N*_RRI^′(*τ*)^PTT^′(*τ*)^_^*m*^(*i*) = the number of **y**(*j*) satisfying the requirement *d*[**x**(*i*), **y**(*j*)]≦*r*, then
    (5) $$\begin{array}{c} C_{{\text{RRI}}^{\prime (\tau )}{\text{PTT}}^{\prime (\tau )}}^{m}\left(i\right)=\frac{N_{{\text{RRI}}^{\prime (\tau )}{\text{PTT}}^{\prime (\tau )}}^{m}\left(i\right)}{N-m+1}. \end{array}$$
    C_RRI^′(*τ*)^PTT^′(*τ*)^_^*m*^(*i*) measures the frequency of the *m*-point PTT^′(*τ*)^ pattern being similar (within a tolerance of ±*r*) to the *m*-point RRI^′(*τ*)^ pattern formed by **x**(*i*).
(4) Average the logarithm of *C*_RRI^′(*τ*)^PTT^′(*τ*)^_^*m*^(*i*) over *i* to obtain *ϕ*_RRI^′(*τ*)^PTT^′(*τ*)^_^*m*^(*r*), as follows:
  (6) $$\begin{array}{c} \phi_{{\text{RRI}}^{\prime (\tau )}{\text{PTT}}^{\prime (\tau )}}^{m}\left(r\right)=\frac{1}{N-m+1}\sum_{i=1}^{N-m+1}\ln C_{{\text{RRI}}^{\prime (\tau )}{\text{PTT}}^{\prime (\tau )}}^{m}\left(i\right). \end{array}$$
(5) Increase *m* by 1, and repeat steps 1 ~ 4 to obtain *C*_RRI^′(*τ*)^PTT^′(*τ*)^_^*m*+1^(*i*), *ϕ*_RRI^′(*τ*)^PTT^′(*τ*)^_^*m*+1^(*r*).
(6) Finally, take Co_ApEn_RRI^′(*τ*)^PTT^′(*τ*)^_(*m*, *r*) = lim⁡_*N*→*∞*_[*ϕ*_RRI^′(*τ*)^PTT^′(*τ*)^_^*m*^(*r*) − *ϕ*_RRI^′(*τ*)^PTT^′(*τ*)^_^*m*+1^(*r*)] and for *N*-point data, the estimate is
  (7) Co_ApEnRRI′(τ)PTT′(τ)(m,r,N)=ϕRRI′(τ)PTT′(τ)m(r) −ϕRRI′(τ)PTT′(τ)m+1(r),
   where *m* represents the chosen vector dimension, *r* represents a tolerance range, and *N* is the data length. To ensure efficiency and accuracy of calculation, the parameters of this study were set at *m* = 3, *r* = 0.15, and *N* = 1000.

#### 2.4.2. RRI and PTT-Based Multiscale Cross-Approximate Entropy Index (MCEI) for Small and Large Scales

The values of Co_ApEn_RRI^′(*τ*)^PTT^′(*τ*)^_(*τ*) were obtained from a range of scale factors between 1 and 20 using the MCE data analysis method. The values of Co_ApEn_RRI^′(*τ*)^PTT^′(*τ*)^_(*τ*) between scale factors 1 and 5 were defined as small scale; those between scale factors 6 and 20 were defined as large scale [23]. The sum of MCE between scale factors 1 and 5 was MCEI_SS_ in (8), while the sum of MCE between scale factors 6 and 20 was MCEI_LS_ in (9). Defining and calculating these two indices of multiscale cross-approximate entropy enables the assessment and quantification of complexity in RRI and PTT between different scale factors. Consider

(8) $$\begin{array}{c} \text{MCE}{\text{I}}_{\text{SS}}=\sum_{\tau =1}^{5}\text{Co}\_\text{ApE}{\text{n}}_{{\text{RRI}}^{\prime (\tau )}{\text{PTT}}^{\prime (\tau )}}\left(\tau \right), \end{array}$$

(9) $$\begin{array}{c} \text{MCE}{\text{I}}_{\text{LS}}=\sum_{\tau =6}^{20}\text{Co}\_\text{ApE}{\text{n}}_{{\text{RRI}}^{\prime (\tau )}{\text{PTT}}^{\prime (\tau )}}\left(\tau \right). \end{array}$$

### 2.5. Multiscale Entropy Index (MEI) Using RRI or PTT Only

Sample entropy (*S*_*E*_) was used to quantify the complexity of RRI or PTT series in twenty scales. The values of *S*_*E*_ between scale factors 1 and 5 were defined as small scale, whereas those between scale factors 6 and 20 were defined as large scale. The sum of MSE in small scale was defined as MEI_SS_, while the sum of MSE in large scale was MEI_LS_ [3].

### 2.6. Statistical Analysis

Average values were expressed as mean ± SD. Significant differences in anthropometric, hemodynamic, and computational parameters (i.e., RRI, PTT, MCEI_SS_, and MCEI_LS_) between different groups were determined using an independent sample *t*-test. Statistical Package for the Social Science (SPSS, version 14.0 for Windows) was used for all statistical analysis. A *P* value less than 0.05 was considered statistically significant.

## 3. Results

### 3.1. Comparison of Basic Demographic and Cardiovascular Parameters in Different Groups

Table 1 presents the basic demographic parameters of Group 1 and Group 2, showing no significant difference in major demographic parameters except for age, HbA1c levels, and body height. Significant differences were observed in body mass index (BMI), waist circumference, systolic blood pressure (SBP), pulse pressure (PP), HbA1c levels, and fasting blood sugar level between Group 2 and Group 3 (Group 3 > Group 2). In addition, significant differences were also observed in HbA1c levels, triglycerides, and fasting blood sugar level between Group 3 and Group 4.

### 3.2. MCEI_LS_ as Parameters Indicative of Age and Diabetic Control

**Figure 2:**
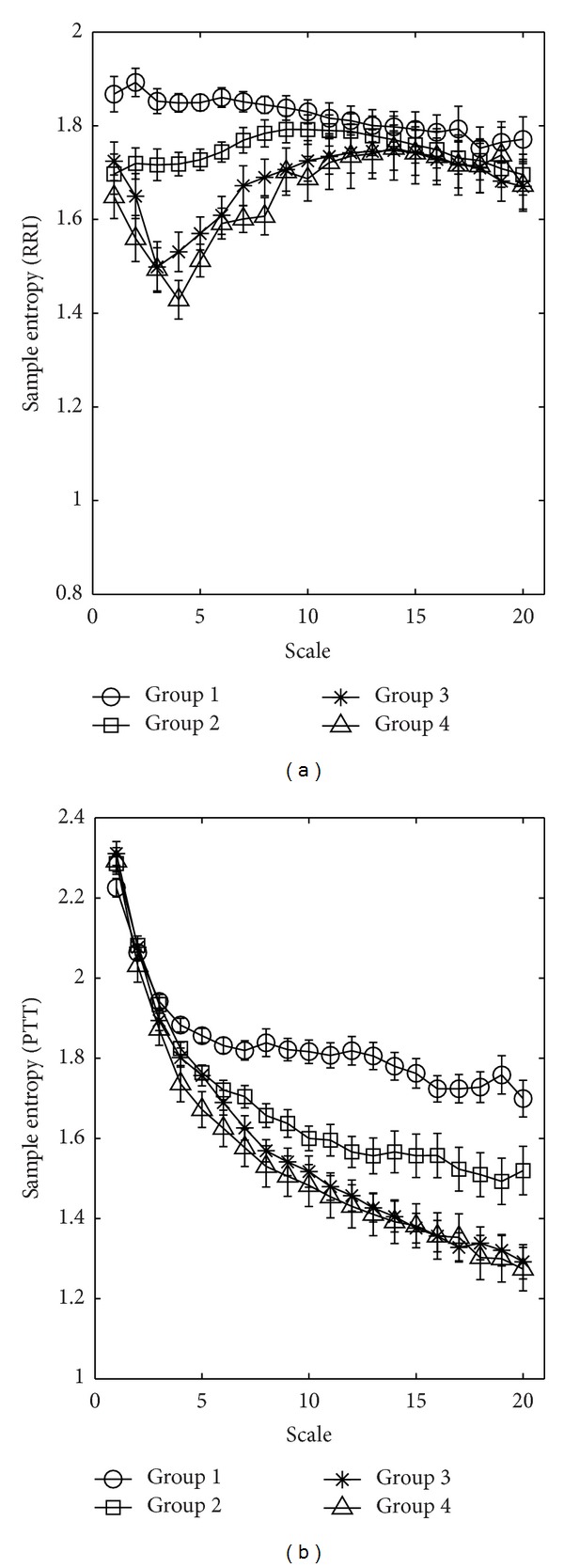
Multiscale entropy (MSE) analysis of (a) RRI and (b) PTT time series showing changes in sample entropy, *S*_*E*_, among the four groups of study subjects for different scale factors. Symbols represent the mean values of entropy for each group, and bars represent the standard error (given by $\text{SE}=\text{SD}/\sqrt{n}$, where *n* is the number of subjects).

There were no significant differences in the values of *S*_*E*_(RRI) and *S*_*E*_(PTT) at any scale (Figure 2), or in MEI_SS_(RRI), MEI_LS_(RRI), MEI_SS_(PTT), and MEI_LS_(PTT) among the 4 groups (Table 1).

**Figure 3:**
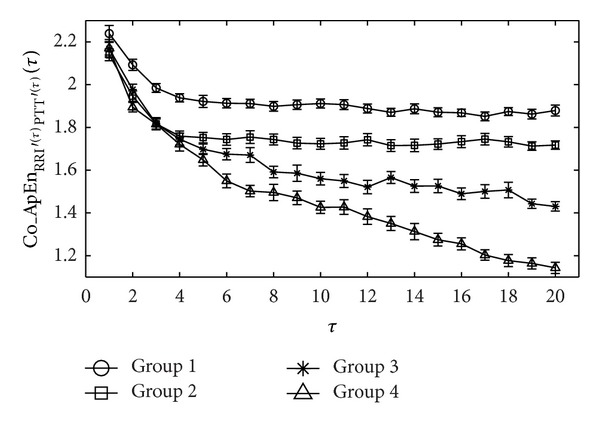
Co_ApEn_RRI^′(*τ*)^PTT^′(*τ*)^_(*τ*) curve of the four groups was calculated using the MCE calculation (*τ* =  1 ~ 20) on 1000 consecutive RRI and PTT times series. Symbols represent the mean values of entropy for each group, and bars represent the standard error (given by $\text{SE}=\text{SD}/\sqrt{n}$, where *n* is the number of subjects).

Figure 3 summarizes the results of the MCE analysis for the values of RRI and PTT time series over 1000 identical cardiac cycles obtained from the four groups of participants. At a scale factor of 1 (*τ* = 1), the magnitudes of Co_ApEn_RRI^′(1)^PTT^′(1)^_(1) ranked as follows: Group 1/Group 3/Group 4/Group 2. The value of Co_ApEn_RRI^′(*τ*)^PTT^′(*τ*)^_(*τ*) began dropping in all groups at a scale factor of 2 (*τ* = 2).

Beginning at a scale factor of 3 (*τ* = 3), the reduction in Co_ApEn_RRI^′(*τ*)^PTT^′(*τ*)^_(*τ*) in Group 1 slowed. However, in the other groups, the values continued decreasing rapidly. Beginning at a scale factor of 5 (*τ* = 5), the Co_ApEn_RRI^′(*τ*)^PTT^′(*τ*)^_(*τ*) of Group 2 achieved stability with only minor fluctuations. The decline in Co_ApEn_RRI^′(*τ*)^PTT^′(*τ*)^_(*τ*) in Group 4 remained greater than that in Group 3. When plotted against large scale factors (i.e., 6–20), the magnitudes of Co_ApEn_RRI^′(*τ*)^PTT^′(*τ*)^_(*τ*) ranked as follows: Group 1, Group 2, Group 3, and Group 4.

MCEI_SS_ only presented a significant difference between Groups 1 and 2 (10.18 ± 0.52 versus 9.42 ± 0.70, *P* < 0.01). The differences among Groups 2, 3, and 4 did not reach statistical significance. In comparison, MCEI_LS_ presented significant differences among all four of the groups (Group 1 versus Group 2: 28.30 ± 1.26 versus 25.96 ± 1.99, *P* < 0.01; Group 2 versus Group 3: 25.96 ± 1.99 versus 23.14 ± 1.85, *P* < 0.01; Group 3 versus Group 4: 23.14 ± 1.85 versus 20.13 ± 1.73, *P* < 0.01) (Table 1).

## 4. Discussion

Since Pincus and Singer's study [19], Co_ApEn has generally been used to reveal similarities between two synchronous, consecutive variables within a single network. This approach has also been used to research the complexity of physiological signals [12, 19]; however, the influence of multiple temporal and spatial scales creates complexity. Thus, this study employed multiscale Co_ApEn (MCE) to evaluate the complexity between the cardiac function-related parameter, RRI, and the atherosclerosis-related parameter, PTT, in the cardiovascular systems of various subject groups.

 Previous studies [1, 2, 18] have also indicated that physiological signals are generally nonlinear and exist in nonstationary states. The use of MSE to quantify complexity within the times series of a single type of physiological signal (i.e., RRI or PWV) demonstrated that the complexity of physiological signals decreases with aging [2] or with the influence of diabetes [3]. In this study, although we used MSE to quantify complexity of RRI or PTT series, there were no significant differences in MEI_SS_(RRI), MEI_LS_(RRI), MEI_SS_(PTT), and MEI_LS_(PTT) between well-controlled and poor-controlled diabetic subjects. Therefore, the influence of the degree of glycemic control on complexity of physiological signals might not be evaluated efficiently according to the use of MSE when analyzing single time series (i.e., RRI or PTT). 

 Drinnan et al.'s study [7] stated that cardiovascular variables such as RRI and PTT are regulated by complex physiological systems and that a strong relationship exists between variations in PTT and those in RRI. We therefore employed the Co_ApEn integrated with preprocessing coarse-graining to calculate MCEI values as well as the complexity between the synchronous time series RRI and PTT. Figure 3 shows that at small-scale factors (from 1 to 5), it is difficult to determine the influence of age, diabetes, or glycemic control based on the complexity between the time series RRI and PTT using Co_ApEn_RRI^′(*τ*)^PTT^′(*τ*)^_(*τ*). Similarly, MCEI_SS_ indicates only that aging reduces the complexity between the two time series. This finding is similar to that of previous studies [3]. As the scale factor increased (from 6 to 20), Co_ApEn_RRI^′(*τ*)^PTT^′(*τ*)^_(*τ*) began revealing significant differences between the four study groups (Figure 3). Table 1 shows that the MCEI_LS_ values of the young healthy subjects were the highest, whereas subjects with poorly controlled type 2 diabetes were the lowest. This may be due to the fact that the coupling effect between the heart and the blood vessels in the cardiovascular system varies according to age and the influence of disease [24, 25]. In other words, the complexity between the time series RRI and PTT decreases due to age and disease.

 Although the MCEI_LS_ can be used to quantify the complexity of RRI and PTT and have been shown to effectively identify significant difference among study groups, limitations still exist. First, a lengthy process of data acquisition and considerable calculation and off-line processing is needed. MCE analysis involves a 30-minute measurement, as opposed to the relatively shorter duration measurement of only RRI and PTT, making the process tiring for participants. The nature of analysis postmeasurement further prevented subjects from receiving their MCEI test results immediately. Second, the medications that the diabetic patients used such as hypoglycemic, antihyperlipidemic, and antihypertensive drugs may also affect autonomic nervous activity. These effects, however, were difficult to assess. The potential effect of medications, therefore, was not considered in the statistical analysis of this study.

## 5. Conclusions

This study integrates cross-approximate entropy with multiple scales to analyze the complexity between two synchronous physiological signals (RRI and PTT) in the cardiovascular system. According to our results, MCEI_LS_ clearly reveals a reduction in the complexity of two physiological signals caused by aging and diabetes.

## Figures and Tables

**Table 1:** Comparisons of demographic, anthropometric, and serum biochemical parameters, MCEI_SS_, and MCEI_LS_ among different subject populations.

Parameters	Group 1	Group 2	Group 3	Group 4
Age, year	26.56 ± 9.60	58.19 ± 8.29**	62.74 ± 0.55	60.58 ± 7.68
Body height, cm	169.38 ± 7.92	162.83 ± 6.85**	161.56 ± 8.97	161.17 ± 7.28
Body weight, kg	66.38 ± 12.21	65.22 ± 11.55	69.40 ± 11.37	73.75 ± 14.86
BMI, kg/m^2^	23.02 ± 3.27	24.55 ± 3.90	26.52 ± 3.21^†^	28.42 ± 5.47
Waist circumference, cm	81.20 ± 11.09	82.94 ± 11.00	93.33 ± 9.37^††^	97.46 ± 3.77
SBP, mmHg	116.50 ± 12.89	115.67 ± 14.12	128.32 ± 16.08^††^	128.46 ± 16.36
DBP, mmHg	71.44 ± 6.70	74.75 ± 9.93	75.58 ± 9.63	78.21 ± 9.89
PP, mmHg	42.97 ± 0.96	40.92 ± 9.29	52.74 ± 14.34^††^	50.25 ± 13.12
HbA1c, %	5.43 ± 0.32	5.84 ± 0.34**	6.74 ± 0.62^††^	9.36 ± 1.59^‡‡^
Triglyceride, mg/dL	88.88 ± 62.54	114.06 ± 88.15	120.87 ± 47.74	168.04 ± 98.43^‡^
Fasting blood sugar, mg/dL	93.13 ± 6.96	97.78 ± 14.69	127.27 ± 24.75^††^	183.96 ± 58.66^‡‡^
MEI_SS_(RRI)	9.31 ± 0.54	8.54 ± 0.78	8.00 ± 1.08^†^	7.64 ± 0.81
MEI_LS_(RRI)	27.11 ± 2.16	26.38 ± 2.07	25.59 ± 2.89	25.45 ± 3.25
MEI_SS_(PTT)	9.97 ± 0.38	9.90 ± 0.40	9.85 ± 0.56	9.50 ± 1.41
MEI_LS_(PTT)	26.73 ± 2.40	23.86 ± 3.71**	21.65 ± 2.55^†^	21.06 ± 4.92
MCEI_SS_	10.18 ± 0.52	9.42 ± 0.70**	9.41 ± 0.62	9.25 ± 0.39
MCEI_LS_	28.30 ± 1.26	25.96 ± 1.99**	23.14 ± 1.85^††^	20.13 ± 1.73^‡‡^

Group 1: healthy young subjects, Group 2: healthy upper middle-aged subjects, Group 3: type 2 diabetic well-controlled patients, Group 4: type 2 diabetic poorly controlled patients. Values are expressed as mean ± SD. BMI: body mass index; SBP: systolic blood pressure; DBP: diastolic blood pressure; PP: pulse pressure; HbA1c: glycosylated hemoglobin; MEI_SS_(RRI): R-R interval-based multiscale entropy index with small scale; MEI_LS_(RRI): R-R interval-based multiscale entropy index with large scale; MEI_SS_(PTT): pulse transit time-based multiscale entropy index with small scale; MEI_LS_(PTT): pulse transit time-based multiscale entropy index with large scale; MCEI_SS_: multiscale Co_ApEn_RRI^′(*τ*)^PTT^′(*τ*)^_(*τ*) index with small scale; MCEI_LS_: multiscale Co_ApEn_RRI^′(*τ*)^PTT^′(*τ*)^_(*τ*) index with large scale.

^†^ *P* < 0.05 Group 2 versus Group 3, ^‡^ *P* < 0.05 Group 3 versus Group 4. **P < 0.01 Group 1 versus Group 2, ^††^ *P* < 0.01 Group 2 versus Group 3, and ^‡‡^ *P* < 0.01 Group 3 versus Group 4.
